# Hemodynamic characterization of Aortic Valve Bypass Surgery (AVBS) using patient-specific computational models based on MRA and PCMR

**DOI:** 10.1186/1532-429X-14-S1-W52

**Published:** 2012-02-01

**Authors:** Adrian Lam, Stephanie Clement-Guinaudeau, Muralidhar Padala, Vinod Thourani, John N Oshinski

**Affiliations:** 1 Georgia Institute of Technology, Atlanta, GA, USA; 2 Emory University, Atlanta, GA, USA

## Background

**Figure 1:**
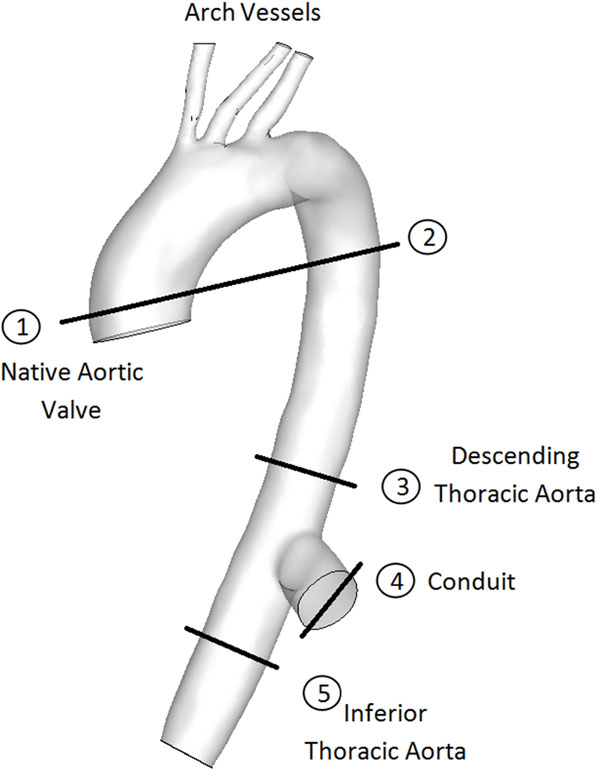
Aortic Valve Bypass Surgery. Lines indicate PCMR image acquisition locations.

AVBS is an option for patients who suffer from both aortic valve stenosis and severe ascending aortic calcification. In AVBS, a conduit containing a prosthetic valve is introduced into the LV transapically and attached to the descending thoracic aorta (Fig 1). However, AVBS patients tend to suffer from a higher number of cerebral events, and intra-aortic thrombus has been reported (Kotani et al, ICVTS, 2009). The objective of this study is to use Magnetic Resonance Angiography (MRA), Phase Contrast Magnetic Resonance (PCMR), and Computational Fluid Dynamics (CFD) to understand the hemodynamics of AVBS in order to correlate how flow patterns may relate to post-surgical complications.

## Methods

20 patients received a follow-up MRI scan 6-12 months after surgery. Contrast-enhanced MRA was used for patient-specific 3D geometry reconstruction. PCMR images were acquired at multiple locations along the descending thoracic aorta and the conduit (Fig 1). Average velocities were obtained from segmented vessels at the native aortic valve and the descending thoracic aorta on PCMR images and used for inlet boundary conditions. Patients with retrograde flow in the descending thoracic aorta (flow from conduit to arch vessels) were selected for modeling. All CFD models were simulated with Fluent ANSYS, Inc. and post-processed using Tecplot.

## Results

**Figure 2:**
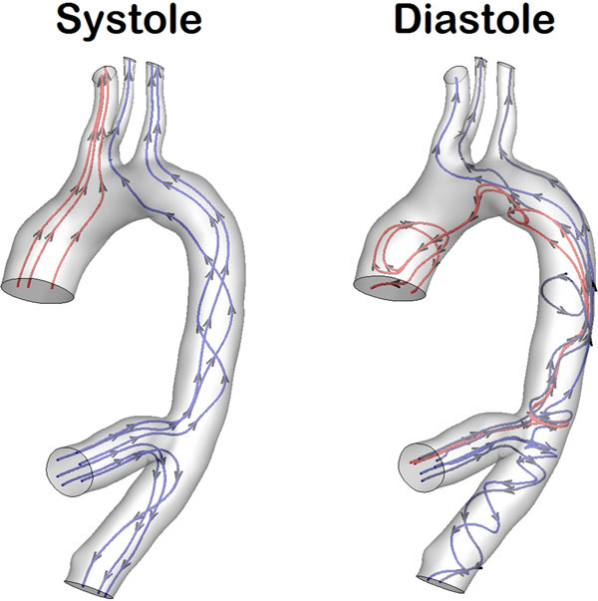
Streamtraces generated from CFD flow field results during systole and diastole. Aortic regurgitation is present during diastole.

Streaklines and streamtraces based on the CFD generated flow field show that the arch vessels are supplied with blood from both the native aorta and conduit. The amount of blood support received from either the native aorta or conduit depends both on vessel geometry and native aortic flow. Oscillatory flow patterns were observed on the inner curvature of the arch, suggesting that the addition of the conduit results in higher vulnerability for intra-aortic plaques leading to thrombosis (Fig 2).

## Conclusions

CFD based on MRA and PCMR can be used to model hemodynamics in AVBS patients. Simulation results indicate that arch vessel perfusion is composed of both native aorta and conduit flow and depends highly on the native aortic flow. CFD results show regions of highly disturbed flow at the inner aortic arch, indicating a possible mechanism for post-surgical complications.

## Funding

This project was funded by the NIH T32 Training Grant.

